# Insights through Genetics of Halophilic Microorganisms and Their Viruses

**DOI:** 10.3390/genes11040388

**Published:** 2020-04-02

**Authors:** Rafael Montalvo-Rodríguez, Julie A. Maupin-Furlow

**Affiliations:** 1 Department of Biology, University of Puerto Rico, Box 9000, Mayagüez, PR 00681, USA; 2 Department of Microbiology and Cell Science, Institute of Food and Agricultural Sciences, University of Florida, Gainesville, FL 32611, USA

**Keywords:** halophile, archaea, bacteria, fungi, virus, genomics, metagenomics, stress, DNA methylation, DNA recombination

## Abstract

Halophilic microorganisms are found in all domains of life and thrive in hypersaline (high salt content) environments. These unusual microbes have been a subject of study for many years due to their interesting properties and physiology. Study of the genetics of halophilic microorganisms (from gene expression and regulation to genomics) has provided understanding into mechanisms of how life can occur at high salinity levels. Here we highlight recent studies that advance knowledge of biological function through study of the genetics of halophilic microorganisms and their viruses.

## 1. Metagenomics

The employment of metagenomics to study microbial diversity and discovering genes with novel functions have proven to be a very powerful tool in microbiology. Considering that halophilic microorganisms present a challenge for this kind of study (mainly because they are understudied and they have a high G+C content), Uritskiy and DiRuggiero [1] present several proposals for the application of these techniques to halophilic microorganisms. The authors explore the limitations and challenges these methodologies currently have and present outlines on how to create better pipelines to study halophilic microbiomes. 

Couto-Rodríguez and Montalvo-Rodríguez [2] used metagenomics to perform a comprehensive temporal study of the microbial community present at the solar salters of Cabo Rojo, Puerto Rico. Their findings revealed that the microbial diversity at genus level of this thalassohaline environment is stable through time, dominated by members of the *Euryarchaeota*, followed by *Bacteroidetes* and *Proteobacteria.* Functional annotation analysis of metagenomic sequences showed a diversity of metabolic genes related to nitrogen fixation, ammonia oxidation, sulfate reduction, sulfur oxidation, and phosphate solubilization. Binning methods allowed the reconstruction of four putative genomes belonging to novel species of Archaea and Bacteria.

## 2. Viruses Genomics

Viruses of halophilic archaea have been a subject of research for years. The obtained knowledge not only provides insights on how infection occurs at high salinity environments, but also it can be useful to develop genetic systems to study halophilic microorganisms. On that line, Dyall-Smith et al. [3] describes the novel myovirus *ChaoS9* which host is *Halobacterium salinarum.* The viral genome consists of a linear dsDNA with approximatedly 55 kb in length. This novel halovirus showed some relationship to PhiH1 and PhiCh1. The genome annotation and organization is also presented. On this direction, Dyall-Smith et al. [4] determined the genome sequence of the halovirus PhiH1 (ΦH1). Eventhough this myohalovirus was discovered in 1982, there is little information about its genome composition. The authors sequenced the genome and present the annotation of the 97 protein coding putative ORFs. 

## 3. Transcriptomics

Transcriptomics studies are extremely useful in understanding the expression and repression of genes in an organism. Tafer et al. [5] combined the tools of genomics and transcriptomics to study the halophilic fungus *Aspergillus salisburgensis*. This organism was isolated at the salt mines of Austria where several extreme conditions exists (high salinity, low nutrient availability and darkness). This fungus was compared to *Aspergillus sclerotialis* which is a halotolerant strain of the genus. Genomic comparison showed several differences specifically at transport-related genes. Differences at gene expression and regulation at the transcriptomic level were also found. The work provides insights on what strategies fungi have develop to grow at extreme conditions specially at high salinity. 

Antisense RNA (asRNA) can function in gene regulation in cells. de Almeida et al. [6] used a transcriptomic approach to map the primary antisense transcriptome of *Halobacterium salinarum* (sp. NRC-1). The researchers found that around 21% of the genes in *Halobacterium salinarum* contain asRNA. A further description of genes possesing this feature is presented as well as comparisons with *Haloferax volcanii* are established.

## 4. Recombination and DNA Modification Systems

Genome sequencing is useful in revealing species with geographic subpopulations, habitat specialization or high frequencies of recombination. With that in mind, Sun et al. [7] analyzed the genome sequences of 25 strains of *Wallemia mellicola*, a xerotolerant and halotolerant fungal species of widespread distribution in indoor and outdoor habitats. From Slovenian chocolate to the hypersaline waters of Spain, the researchers found the *W. mellicola* genome sequences to be relatively homogenous with no apparent clusters of strains based on habitat or geographic location. The authors suggest that *W. mellicola* strains undergo a reasonable amount of recombination shuffling between genomes of individual organisms and likely do this via sexual reproduction. This suggestion is based on phylogenetic analysis of core Benchmarking Universal Single-Copy Orthologues (BUSCOs), the density of single nucleotide polymorphisms (SNPs) and the identification of putative mating-type loci. 

DNA methyltransferases (MTases) and restriction modification (RM) systems are important in a variety of functions including restricting foreign genomes and host DNA repair by recombination. In this special issue, Fullmer et al. [8] provide a survey of the distribution of RM system and orphan MTase gene homologs among halophilic Archaea of the class *Halobacteria*. One striking result was the irregular distribution of RM system candidate genes among the orders, genera, species, and even communities and populations of the *Halobacteria*. Based on this patchy distribution, the authors suggest that the RM systems are selfish genetic elements that undergo frequent horizontal gene transfer and gene loss. By contrast, the orphan MTase gene homologs were highly conserved and, thus, appeared functionally constrained among the *Halobacteria* lineages. Under-(CTAG) and over-(GATC) represented motifs were also identified in the genome sequences that may be targets of the MTase and RM systems.

## 5. Metabolism and Stress Responses

Halophilic microorganisms are considered a resource for industrial catalysts that function in organic solvent, high salt or other extreme conditions that most organisms cannot tolerate. Liao et al. [9] provide insight into an anaerobic haloalkaliphilic bacterium, *Alkalitalea saponilacus*, that uses xylan as a sole carbon and energy source and produces propionic acid as a major product. Microbes and the enzymes that hydrolze xylan (xylanases) are useful in the biobleaching of wood pulp as well as in the depolymerization of lignocellulosic biomass to generate renewable fuels and chemicals. In this special issue, the authors [9] find *A. saponilacus* secretes an extracellular fraction that hydrolyzes xylan in high salt and, through genomic sequencing, identify gene homologs relating to the pathway for complete xylan degradation. One future aim of this work is to develop a method to recover the xylanase for use in biobleaching wood pulp. Now that the genome sequence is available, genetic engineering may be an option for enhancing production of this halotolerant xylanase.

Halophilic archaea are masters at handling stress as these microbes thrive in hypersaline environments that promote hyperosmotic shock, desiccation, high UV exposure and other extreme factors. By screening a transposon mutant library of *Haloferax volcanii*, Gomez et al. [10] probed the molecular factors responsible for oxidative stress response. Transposon mutants hypertolerant of oxidant were isolated and found to have insertions at loci associated with post-translational modification, transport, polyamine biosynthesis, electron transfer and other cellular processes. As follow-up by markerless deletion, the authors demonstrated that cells producing 20S proteasomes of α2 and β (and not α1) subunits were more tolerant of oxidative stress than wild type. Thus, modulation of the subunit composition of one of the central proteolytic systems of these microbes (i.e., proteasomes) appears important in stress response. 

Halophile genetics can provide understanding into the function of unusual groups of proteins as evidenced by the work of Nagel et al. [11]. In this work, the researchers examined the function of ORFs predicted to encode small proteins of less than 100 amino acids that harbor a zinc finger motif (Cys/His pattern of two Cys or His residues separated by two to three intermediate amino acids). Through systematic and targeted deletion, the researchers identified 12 ORFs encoding putative zinc finger proteins that were correlated with the ability of cells to adapt to stress, form biofilms and/or swarm. This type of approach offers a strong foundation for future studies to reveal how these zinc finger proteins may interact with DNA, RNA, proteins, lipids, and/or small molecules to alter the biological function of the cell.
